# Clinical characteristics and severity of concomitant atopic dermatitis in adults with asthma: a nationwide population-based registry study

**DOI:** 10.3389/falgy.2026.1826356

**Published:** 2026-06-11

**Authors:** Amalie Thorsti Møller Rønnstad, Zarqa Ali, Kjell Erik Julius Håkansson, Mia-Louise Nielsen, Lea Krog Nymand, Simon Francis Thomsen, Alexander Egeberg, Charlotte Suppli Ulrik, Jacob P. Thyssen

**Affiliations:** 1 Department of Dermatology, Bispebjerg Hospital, University of Copenhagen, Copenhagen, Denmark; 2 Department of Respiratory Medicine, Copenhagen University Hospital, Hvidovre, Denmark; 3 Department of Biomedical Sciences, University of Copenhagen, Copenhagen, Denmark; 4 Department of Clinical Medicine, University of Copenhagen, Copenhagen, Denmark

**Keywords:** asthma, asthma severity, atopic dermatitis, atopic dermatitis severity, GINA, PO-SCORAD

## Abstract

**Background:**

Atopic dermatitis (AD) often coexists with asthma, but the characteristics of AD in adults with asthma are largely unknown.

**Objective:**

The objective of this work was to examine the characteristics and severity of AD in adults with asthma.

**Methods:**

This population-based registry study identified adults (18–45 years) with an inpatient or outpatient asthma diagnosis in Danish hospitals between 1 January 2000 and 31 December 2019. AD history was determined by inpatient or outpatient diagnostic codes. AD and asthma severity were assessed through patient surveys using the Patient-Oriented SCORing Atopic Dermatitis (PO-SCORAD) and the Global Initiative for Asthma (GINA) 2024.

**Results:**

Among 4,126 patients (15% response rate), 5.2% had AD. Of these, 22.7% were treated with GINA Step 4–5. Patients with AD were primarily women (74.3%) with childhood-onset AD (90.7%) and asthma (82.6%, *p* < 0.001). Mean (standard deviation) PO-SCORAD was 25.4 (16.1)). Asthma severity was not correlated or associated with AD severity. AD involvement in the head-and-neck region and on hands was common (54.0%; 61.3%) and associated with higher PO-SCORAD scores (*p* < 0.05; *p* < 0.001). AD flares during asthma exacerbations were reported by 33.0% of patients (*p* < 0.001).

**Conclusions:**

Asthma patients with AD often have childhood onset of both conditions and mild-to-moderate AD. Clinicians should recognize that AD in high-burden skin areas is common in asthma patients.

## Highlights

- Adult asthma patients with concomitant atopic dermatitis (AD) primarily reported childhood onset of AD and mild-to-moderate disease.
- Childhood-onset asthma was associated with a history of AD, but asthma severity and AD severity were not correlated.
- AD in the head-and-neck area and on the hands was common in asthma patients and associated with a higher PO-SCORAD score. AD flares during asthma exacerbations were also common.

## Introduction

Asthma and atopic dermatitis (AD) are common chronic inflammatory diseases that share immunopathogenic mechanisms and represent some of the most frequent presentations of type 2 inflammation in the airways and skin (1, 2). Asthma is characterized by wheezing, dyspnea, and coughing with an estimated lifetime prevalence of 17.0% in Denmark (3). In up to 50% of cases, asthma symptoms emerge during childhood (4), frequently following the onset of AD (5). About 50% of children diagnosed with AD before 2 years of age develop asthma, in part due to allergic sensitization caused by allergens getting easily absorbed through the impaired skin barrier (6). In asthma, hyperresponsiveness of the airways often has an underlying type-2 (Th2) immune response (7), driven in part by the release of interleukin (IL)-4, IL-5, and IL-13, in turn promoting eosinophilic inflammation, immunoglobulin E production, mucus hypersecretion, and airway remodeling (8–10). AD is characterized by eczema that is waxing and waning in typical sites, depending on age (2, 11, 12), and affects up to 20%–25% of infants and 3%–7% of adults across the world (2). Around 90% of AD cases present before the age of 5 years (12, 13). Children with AD have more than a 2-fold increased risk of developing asthma, highlighting the shared immunopathogenesis (2, 14). A systematic review and meta-analysis estimated the pooled prevalence of asthma to be 25.7% in AD patients (15). Limited data exist on the prevalence of AD in asthma patients (16, 17), and no studies have examined the clinical characteristics of AD in patients with asthma. Therefore, we conducted this registry-based survey to evaluate the clinical characteristics and severity of AD among adults with asthma and explored possible correlations with asthma severity.

## Material and methods

The study was approved by the Danish Data Protection Agency (P-2020-1016-13083). All participants provided informed written consent for processing of personal information.

### Study design

Adult Danes (≥18–45 years) seen at Danish hospitals with an inpatient or outpatient asthma diagnosis [International Classification of Diseases-10 (ICD-10) code J45.X] between 1 January 2000 and 31 December 2019 were identified through the Danish National Patient Register (NPR) (18). The age range was limited to 18–45 years to reduce potential misclassification related to chronic obstructive pulmonary disease. Asthma patients were invited to participate in a survey on atopic disease. Data were collected using the secure platform Research Electronic Data Capture (REDCap), hosted by the Capital Region of Denmark. In March 2024, invitations were distributed through e-Boks, a secure digital mailbox used by the Danish government and official institutions to communicate with citizens aged >15 years. Hospital-recorded AD was defined as an inpatient or outpatient hospital diagnosis for AD (ICD-10 code L20.X) registered in the NPR during the patient’s lifetime.

### Clinical parameters

Self-reported patient characteristics were collected along with current asthma signs and symptoms, medication use, disease severity, and control. Signs and symptoms of AD were assessed in patients with concomitant AD (Supplementary Table S1).

Current self-reported asthma medication (past 4 weeks) was classified according to the Global Initiative for Asthma (GINA) 2024, track 1/2 guidelines (4). GINA Steps 1–2 corresponded to mild asthma, GINA Step 3 to moderate, GINA Step 4 to moderate-to-severe, and GINA Step 5 to severe (4). Asthma control was evaluated using three proxies: use of systemic prednisolone for asthma (past 12 months), daily asthma symptoms (>2 days per week as defined by GINA), and Asthma Control Questionnaire-5 (ACQ-5) scores. For patient-reported symptom burden, *well-controlled* asthma was defined as a score <0.75. A score of 0.75–1.25 indicated *partially controlled*, while a score >1.25 defined *uncontrolled* (19). Prescriptions of topical corticosteroids (TCS) or topical calcineurin inhibitors (TCI) within last 12 months were identified through the Danish National Prescription Registry (20).

Current severity and anatomical site of AD were assessed using the Patient-Oriented SCORing Atopic Dermatitis (PO-SCORAD) (21). Mild AD was defined as a score <25, moderate 25–50, and severe >50. The Patient-Oriented Eczema Measure (POEM) measured subjective severity (22). Clear or almost clear AD was defined as a score between 0 and 2, mild 3–7, moderate 8–16, severe 17–24, and very severe 25–28 (23).

### Statistical analysis

Analyses were performed using RStudio V1.4.1717 and R V4.1.2. Categorical variables were presented as frequencies. For continuous variables with normal distribution, mean and standard deviation (SD) were calculated and for variables that were non-normally distributed, median and interquartile range (IQR) were calculated. To correct for multiple testing and reduce the risk of type I errors, the false discovery rate was used. Logistic regression examined characteristics associated with AD. Fully adjusted models included age, sex, educational level, asthma onset, and ACQ-5. Sensitivity analyses replaced ACQ-5 with systemic prednisolone for asthma (last 12 months) or daily asthma symptoms (>2 days per week). Characteristics were chosen *a priori*, and estimates were reported as adjusted odds ratios (aOR) with 95% confidence intervals (CIs). Spearman's rank correlation assessed the associations between AD severity and asthma severity and control. Multiple linear regression examined PO-SCORAD-associated characteristics. Asthma-related predictors included asthma severity, asthma onset, and ACQ-5. AD characteristics included involvement of high-burden areas, foot involvement, and AD onset; analyses were adjusted for AD-affected body surface area (BSA). In sensitivity analyses with AD as the outcome, an ICD-10 code of AD was replaced with a prescription of TCS/TCI the previous 12 months. Associations between AD flares during asthma exacerbations and PO-SCORAD were examined in a separate BSA-adjusted model. All models were adjusted for age and sex, and results presented as adjusted coefficients with 95% CI.

## Results

### Demographics

A total of 4,126 adults participated (response rate 15%), with higher response among women (16.3%) than men (12.5%). Women constituted 70.3% of the cohort, and the mean (SD) age was 37.1 years (5.5). Most participants were women >30 years (58.6%), followed by men >30 years (26.2%). The cohort was representative of the invited population in terms of age [mean (SD) age 36.6 (5.5) years], but men were underrepresented (Supplementary Table S2).

### Clinical characteristics of asthma

Of the patients, 63.5% (*n* = 2,319) reported childhood-onset asthma. Asthma symptoms >2 days per week were reported by 24.5% (*n* = 870). According to GINA guidelines, 22.7% had moderate-to-severe asthma. Among asthma patients, 37.5% used inhaled corticosteroid/Long-acting beta_2_-agonist (ICS/LABA), 60.9%% reported *well-controlled* asthma, and 12.0% reported systemic prednisolone use (past 12 months) (Table 1, Supplementary Table S3).

**Table 1:** Baseline characteristics of asthma patients with and without atopic dermatitis.

		Asthma patients with AD (*n* = 214)	Asthma patients without AD (*n* = 3,912)	All asthma patients (*n* = 4,126)
Age, mean (SD)		**36.8** (5.5)	**37.1** (5.5)	**37.1** (5.5)
Female, % (*n*/*n* total)		**74.3** (159/214)	**70.0** (2,740/3,912)	**70.3** (2,899/4,126)
Educational level, % (*n*/*n* total)	Low	**20.7** (38/184)	**22.1** (762/3,454)	**22.0** (800/3,638)
	Middle	**51.6** (95/184)	**49.6** (1,713/3,454)	**49.7** (1,808/3,638)
	High	**27.7** (51/184)	**28.3** (979/3,454)	**28.3** (1,030/3,638)
BMI, median (IQR)		**25.6** (22.6–28.7)	**25.8** (22.8–30.1)	**25.8** (22.8–30.1)
Ever-smokers, % (*n*/*n* total)		**37.8** (71/188)	**38.4** (1,364/3,552)	**38.4** (1,435/3,740)
Pack years, mean (SD)		**7.1** (5.8)	**7.8** (7.9)	**7.8** (7.8)
Physical activity h/week, % (*n*/*n* total)	0–2	**38.3** (72/188)	**44.5** (1,580/3,554)	**44.1** (1,652/3,742)
	3–6	**50.0** (94/188)	**45.6** (1,620/3,554)	**45.8** (1,714/3,742)
	Above 7	**11.7** (22/188)	**10.0** (354/3,554)	**10.0** (376/3,742)
Asthma characteristics				
Age at asthma onset, % (*n*/*n* total)	Childhood	**82.6** (152/184)	**62.5** (2,167/3,469)	**63.5** (1,334/3,653)
	Adult	**17.4** (32/184)	**37.5** (1,302/3,469)	**36.5** (1,334/3,653)
Asthma symptoms last 12 months, % (*n*/*n* total)		**71.3** (127/178)	**70.7** (2,384/3,370)	**70.8** (2,511/3,548)
Asthma severity (GINA 2024), % (*n*/*n* total)	Step 1–2	**37.7** (66/175)	**40.5** (1,369/3,379)	**40.4** (1,435/3,554)
	Step 3	**38.9** (68/175)	**36.8** (1,244/3,379)	**36.9** (1,312/3,554)
	Step 4	**15.4** (27/175)	**13.6** (459/3,379)	**13.7** (486/3,554)
	Step 5	**8.0** (14/175)	**9.1** (307/3,379)	**9.0** (321/3,554)
ACQ-5, median (IQR)		**0.4** (0.0–1.0)	**0.4** (0.0–1.2)	**0.4** (0.0–1.2)
ACQ-5 *well controlled asthma*, % (*n*/*n* total)		**65.9** (112/170)	**60.7** (2,011/3,315)	**60.9** (2,123/3,485)
Daily asthma symptoms >2 days a week, % (*n*/*n* total)		**17.7** (31/175)	**24.9** (840/3,376)	**24.5** (871/3,551)
Asthma-related night awakening last 4 weeks, % (*n*/*n* total)		**11.5** (20/174)	**11.9** (400/3,375)	**11.8** (420/3,549)
Emergency room or hospitalization last 12 months, % (*n*/*n* total)		**3.9** (5/127)	**6.7** (159/2,374)	**6.6** (164/2,501)
Prednisolone treatment for asthma last 12 months, % (*n*/*n* total)		**11.1** (14/126)	**12.1** (279/2,304)	**12.1** (293/2,430)
AQLQ score, median (IQR)		**6.6** (6.1–6.8)	**6.5** (5.8–6.8)	**6.5** (5.8–6.8)

ACQ-5, Asthma Control Questionnaire 5; AD, atopic dermatitis; AQLQ, Asthma Quality of Life Questionnaire; BMI, body mass index; GINA, global initiative for asthma; h, hours; ICD-10, International Classification of Diseases; IQR, interquartile range; *n*, number; SD, standard deviation.

a Low = high school diploma or vocational school, Middle = bachelor's degree, and High = master's degree.

The bold values indicate both be % value, mean or median values.

### Prevalence of atopic dermatitis

Among asthma patients, 5.2% (*n* = 214) had hospital-recorded AD. Stratified by asthma severity, no increase in AD prevalence was observed as severity increased (Supplementary Figure 1). Women and men exhibited comparable prevalences (5.5% and 4.5%, respectively). Age stratification revealed no consistent decline in prevalence with age, but patients between 43 and 45 years had the lowest prevalence (Supplementary Figure 1).

#### TCS and TCI in the previous 12 months

Among asthma patients, 15.7% (*n* = 646) received a prescription for TCS and/or TCI within the past 12 months. Prescription frequency did not increase with asthma severity (mild 20.8%; moderate 16.2%; moderate-to-severe 17.5%; severe 15.6%).

### Clinical characteristics of patients with and without concomitant atopic dermatitis

Mean (SD) age was comparable between asthma patients with and without AD [36.8 (5.5) vs. 37.1 (5.5) years], and the majority were women (74.3% and 70.0%). Childhood-onset asthma was more frequent in patients with concomitant AD than those without (82.6% vs. 62.5%, *p* < 0.001). More patients with AD reported dupilumab treatment for AD and asthma than patients with only asthma [4.7% (10/214) vs. 0.20% (8/3,912), *p* < 0.001]. Asthma patients with concurrent AD predominantly had mild or moderate asthma (37.7% and 38.9%) and showed no significant differences in asthma severity or control (ACQ-5) compared with those without AD (Table 1). The prevalence of daily asthma symptoms was higher among asthma patients without AD than those with (*p* < 0.001) (Table 1).

#### Association between clinical characteristics and atopic dermatitis

Asthma severity (GINA) was not associated with concomitant AD in any analyses, including models adjusted for age, sex, education, childhood-onset asthma, and ACQ-5. Childhood-onset asthma was associated with concomitant AD, with increased odds in fully adjusted models [aOR = 3.68 (95% CI, 2.40–5.91)]. Poor asthma control (ACQ-5) was not associated with AD (Table 2). Replacing ACQ-5 with systemic prednisolone use (last 12 months) did not demonstrate associations with concomitant AD. However, patients without AD had higher odds of daily asthma than those with AD [aOR = 1.56 (95% CI, 1.03–2.42)]. In sensitivity analyses with AD as the outcome, replacement of an ICD-10 code of AD with a prescription of TCS/TCI in the previous 12 months, showed no association between any characteristics and a prescription the last 12 months (Supplementary Table S6).

**Table 2:** Characteristics associated with hospital-recorded AD, adjusted odds ratio, and 95% confidence interval.

Atopic dermatitis characteristics		Hospital-recorded AD, aOR [95% CI]		
		Model 1^a^	Model 2^b^	Model 3^c^
Age		0.99 [0.97–1.03]	1.01 [0.98–1.04]	1.00 [0.97–1.03]
Sex	Men	Ref	Ref	Ref
	Women	1.18 [0.83–1.71]	1.29 [0.90–1.86]	1.30 [0.91–1.90]
Educational level	Low	Ref	Ref	Ref
	Middle	1.04 [0.69–1.58]	1.03 [0.68–1.57]	1.05 [0.69–1.63]
	High	1.05 [0.67–1.65]	1.04 [0.67–1.65]	1.04 [0.65–1.67]
Asthma severity, GINA	Mild	Ref	Ref	Ref
	Moderate	1.15 [0.81–1.63]	1.15 [0.81–1.63]	1.19 [0.83–1.72]
	Moderate-to-severe	1.08 [0.66–1.73]	1.12 [0.68–1.79]	1.24 [0.74–2.01]
	Severe	0.89 [0.46–1.58]	0.95 [0.49–1.69]	1.09 [0.55–1.99]
Onset of asthma	Childhood		3.63 [2.39–5.77]*	3.68 [2.40–5.91]*
	Adult		Ref	Ref
ACQ-5 score	Uncontrolled			0.83 [0.58–1.17]
	Well controlled			Ref

aOR, adjusted odds ratio; ACQ-5, the Asthma Control Questionnaire-5; AD, atopic dermatitis; GINA, Global Initiative for Asthma; 95% CI, 95% confidence interval.

Logistics regression models showing clinical characteristics associated with hospital-recorded AD in asthma patients.

a In model 1, age (continuous), sex (men/women), educational level (low/middle/high), and asthma severity according to GINA (mild/moderate/moderate-to-severe/severe) are included as explanatory variables.

b In model 2, age (continuous), sex (men/women), educational level (low/middle/high), asthma severity according to GINA (mild/moderate/moderate-to-severe/severe), and age of asthma onset (childhood/adulthood) are included as explanatory variables.

c In model 3, age (continuous), sex (men/women), educational level (low/middle/high), asthma severity according to GINA (mild/moderate/moderate-to-severe/severe), age of asthma onset (childhood/adulthood), and asthma control according to the ACQ-5 score (not well controlled/well controlled) are included as explanatory variables.

* *p* < 0.001.

### Atopic dermatitis characteristics in asthma patients

Seventy-eight percent (*n* = 117) of asthma patients with hospital-recorded AD had current AD symptoms. Childhood onset of AD was common, with 90.7% (*n* = 136) reporting it and 83.8% of those also reporting childhood-onset asthma. Among patients who reported adult onset of AD, 78.6% (*n* = 11) also reported childhood-onset asthma. AD flares during asthma exacerbation were reported by 33.0% (*n* = 32), and of these patients, 46.9% (15/32) had uncontrolled asthma (ACQ-5) compared to only 26.2% (17/65) who did not report AD flares during asthma exacerbations. However, the difference was not statistically significant (Fisher's exact *p* = 0.065). AD in high-burden areas such as the head-and-neck region, the hands, and the genitals was reported by 54.0%, 61.3%, and 3.3%, respectively. Furthermore, 14.7% had foot involvement. Additional characteristics are presented in Table 3.

**Table 3:** Atopic dermatitis-related characteristics in adult patients with asthma.

Atopic dermatitis characteristics		Asthma patients with hospital-recorded AD (*n* = 214)
Age at AD onset, % (*n*/*n* total)	Childhood	**90.7** (136/150)
	Adult	**9.3** (14/150)
Current AD symptoms, % (*n*/*n* total)		**78.0** (117/150)
Consulted a doctor for AD last 12 months, % (*n*/*n* total)	1–2 times	**21.1** (31/147)
	≥3 times	**12.9** (19/147)
Flare of AD during asthma exacerbation, % (*n*/*n* total)		**33.0** (32/97)
Foot eczema, % (*n*/*n* total)		**14.7** (22/150)
AD involvement of high-burden skin areas, % (*n*/*n* total)	Hand eczema	**61.3** (92/150)
	Genital eczema	**3.3** (5/150)
	Head-and-neck dermatitis	**54.0** (81/150)
Head-and-neck dermatitis severity, mean (SD)^a^		**4.0** (2.4)
PO-SCORAD, mean (SD)		**26.8** (15.3)
PO-SCORAD, % (*n*/*n* total)	Clear	**5.3** (8/150)
	Mild	**45.3** (68/150)
	Moderate	**42.0** (63/150)
	Severe	**7.3** (11/150)
POEM, mean (SD)		**10.8** (5.8)
POEM, % (*n*/*n* total)	Clear or almost clear	**21.9** (32/146)
	Mild	**20.5** (30/146)
	Moderate	**45.2** (66/146)
	Severe-very severe	**12.3** (18/146)
DLQI, median (IQR)		**4.0** (2.0–6.0)
Current AD treatment, % (*n*/*n* total)	TCS	**63.3** (95/150)
	TCI	**22.0** (33/150)
	Other systemics^b^	**6.0** (9/150)
	Dupilumab	**6.0** (9/150)

AD, atopic dermatitis; DLQI, Dermatology Life Quality Index; ICD-10, International Classification of Diseases; IQR, interquartile range; *n*, number; POEM, Patient-Oriented eczema Measure; PO-SCORAD, Patient-Oriented SCORing Atopic Dermatitis; SD, standard deviation; TCI, topical calcineurin inhibitors; TCS, topical corticosteroids.

a Current severity of head-and-neck dermatitis measured on a scale from 0 to 10 (0 = mildest, 10 = most severe).

b Treatment with methotrexate, prednisolone tablets, azathioprine, or cyclosporine.

#### Atopic dermatitis severity

**Figure 1:**
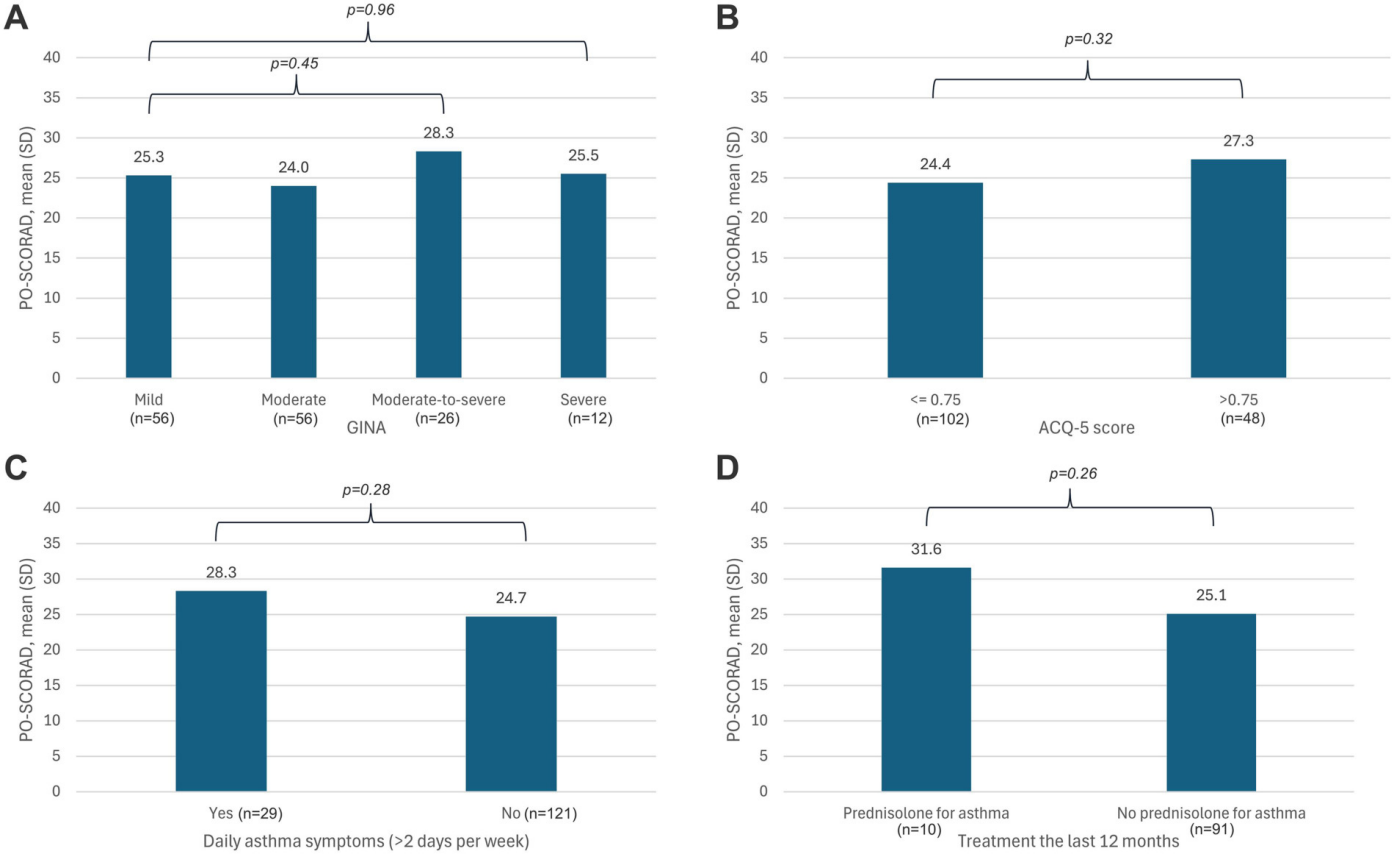
Mean (SD) AD severity (PO-SCORAD) stratified by asthma severity and control parameters in asthma patients. ACQ-5, the Asthma Control Questionnaire-5; AD, atopic dermatitis; ICD-10, the International Classification of Diseases 10th; GINA, Global Initiative for Asthma; PO-SCORAD, Patient-Oriented SCORing Atopic dermatitis; SD, standard deviation. Mean (SD) PO-SCORAD stratified by **(A)** GINA, **(B)** ACQ-5 score, **(C)** prednisolone treatment over the past 12 months for asthma, and **(D)** asthma-related night awakenings over the past 4 weeks in asthma patients with a history of hospital-recorded AD (ICD-10, L20.X).

Asthma patients with concomitant AD had a mean (SD) PO-SCORAD of 25.4 (16.1), with 45.3% and 42.0% having mild and moderate AD, respectively. The mean (SD) POEM score was 9.0 (6.7), with 45.2% reporting moderate AD. Across asthma characteristics, PO-SCORAD was highest in patients who reported AD flares during asthma exacerbations [38.6 (13.5)] and those who used systemic prednisolone (past 12 months) [31.6 (16.9)] (Figure 1). The mean (SD) PO-SCORAD was not significantly different between men and women or in patients ≤30 and >30 years. PO-SCORAD did not increase with increasing asthma severity (Supplementary Table S4).

Asthma patients with childhood-onset AD had numerically higher PO-SCORAD compared to adult-onset patients, but the difference was not statistically significant (Supplementary Table S4). Patients with AD in high-burden areas demonstrated higher PO-SCORAD than those without, with mean (SD) values of 33.0 (14.8) versus 16.4 (11.8) for head-and-neck dermatitis (HND) (*p* < 0.01), and 31.1 (14.4) versus 16.2 (14.4) for HE (*p* < 0.001). Patients with genital AD had higher scores than those without at 36.3 (18.3) versus 25.0 (18.3), but the difference was not statistically significant (*p* = 0.16). Patients with foot involvement yielded the highest PO-SCORAD, with 38.4 (15.0) compared with 23.1 (15.2) (*p* < 0.001) in patients without foot involvement.

#### Association between atopic dermatitis severity and asthma severity and control

Asthma severity (GINA) and AD severity (PO-SCORAD) were not correlated (*r* = 0.02, *p* = 0.51). Asthma control (ACQ-5) and AD severity (PO-SCORAD) showed a weak correlation (*r* = 0.21, *p* < 0.001). POEM demonstrated the same tendency, with no correlation with GINA, and a weak correlation with ACQ-5 (*r* = 0.15, *p* < 0.001). PO-SCORAD was not associated with asthma severity (GINA), ACQ-5, childhood-onset asthma, systemic prednisolone for asthma (last 12 months), or daily asthma symptoms (>2 days per week) in adjusted analyses. The findings remained unchanged after stratification for age and sex (not shown).

#### Atopic dermatitis characteristics associated with atopic dermatitis severity

In fully adjusted analyses, increasing AD severity (PO-SCORAD) was associated with AD involvement of the head-and-neck region and the hands [*β* = 5.94 [95% CI, 0.34–11.60] and *β* = 8.37 [95% CI, 4.07–12.70]]. After adjustment, PO-SCORAD was not associated with genital or foot involvement (Supplementary Table S5). AD flares during asthma exacerbation were associated with higher PO-SCORAD after adjustment for age, sex, and BSA.

## Discussion

### Main findings

In this registry-based survey of adult asthma patients, 5.2% had a history of hospital-recorded AD and 15.7% had used TCS and/or TCI. Most AD cases were diagnosed in childhood along with asthma. Patients with and without AD had similar asthma severity, but patients without AD had more daily asthma symptoms. Most asthma patients with AD were women and had mild-to-moderate AD. Involvement of the head-and-neck region and the hands was common and associated with higher AD severity. Asthma and AD severity were not correlated or associated, but reports of AD flares during asthma exacerbation were associated with higher PO-SCORAD scores.

### Interpretation

A history of hospital-recorded AD was observed in 4.4%–5.6%, and TCS and/or TCI had been used by 15.6%–20.8% of adults across mild-to-severe asthma. Most studies have evaluated asthma prevalence among AD patients, with a meta-analysis reporting an asthma prevalence of 25.7% in AD patients (15). In contrast, few studies have examined AD prevalence in asthma patients. For example, an ISAR-based study reported that 10% of 11,821 patients with severe asthma (GINA) had AD based on physician diagnosis (16). In Danish studies, the AD lifetime prevalence varies by ascertainment method in the general population. Estimates range from 4.2%–16.2% depending on the combination of UK Working Party criteria applied (24), 0.5% based on ICD–10 codes (L20.X) in the registries (25), and 9.0% in a recent questionnaire study (26). A US study estimated the AD prevalence to be 7.3% across 1,278 adults (27), and a Swedish study of 34,313 adults found 14% to have AD (28). In the present study, AD was also identified using hospital records (L20.X), a method with a positive predictive value of 95% but low sensitivity (29). Compared to Danish registry data from the general population, the AD prevalence of 5.2% in asthma patients is high. However, the Danish NPR does not capture AD managed by private dermatologists, general practitioners, or undiagnosed patients not seeking medical care, potentially underestimating the prevalence. Use of TCS and/or TCI is not a direct AD measure; nevertheless, the high prevalence of prescriptions suggests a substantial burden of skin disease among asthma patients. However, sensitivity analyses demonstrated no association between childhood-onset asthma and AD when defined by TCS and/or TCI prescriptions within the previous 12 months, suggesting that ascertainment of AD based on the ICD-10 code L20.x may provide a more robust and specific measure of disease status, particularly given the well-established comorbidity between AD and asthma in childhood.

Childhood-onset asthma was associated with concomitant AD, and 90.7% of patients with AD reported childhood-onset AD. Recall bias may affect this number, as Danish and Swedish studies found that 29% and 44% of people, respectively, did not recall having childhood AD when surveyed in adulthood (30, 31). Nonetheless, the high proportion may partly reflect a chronic course persisting into adolescence and adulthood, with greater disease awareness. This proportion exceeds that reported from Danish data of 6,716 adult AD patients, where 72.1%–81.4% had childhood-onset AD (32). Similarly, a meta-analysis found that 26% had adult onset of AD (33). Early-onset AD, especially when persistent or severe, has been shown to increase the likelihood of asthma (34, 35). Thus, adult asthma cohorts may contain a higher proportion of early-onset AD, reflecting the accumulation of atopic disease and possible trajectory of an atopic march (36).

Concomitant AD was mainly of mild-to-moderate severity in asthma patients. The mean (SD) PO-SCORAD and POEM scores were 25.4 (16.1) and 9.0 (6.7), respectively, with 42.0% and 45.2% classified as moderate disease. These estimates are comparable to the study of 6,716 AD adults from the same registry, in which 14.0–50.8% (PO-SCORAD) and 11.5–44.0% (POEM) had moderate disease (32). This suggests that AD severity among asthma patients with concomitant AD is comparable to that seen in broader AD cohorts, where a proportion of patients also have concomitant asthma. This coexistence reflects the interplay between the diseases, highlighting that shared pathophysiology contributes to their concurrent presentation. As AD was mild to moderate, this group may be overlooked in respiratory care, potentially delaying referral and optimal dermatological treatment.

Even though asthma severity and AD severity were not correlated or associated, 33.0% reported AD flares during asthma exacerbations, and these patients had higher PO-SCORAD scores in adjusted analyses. AD flares and asthma exacerbations have been scarcely investigated, but a UK registry study showed that asthmatic patients with AD had an increased risk of exacerbations, with the risk rising with AD severity (37). AD flares may reflect increased systemic type 2 inflammation, with cytokines such as IL-4, IL-13, and IL-5 contributing to both skin barrier dysfunction and airway inflammation (9, 38–40). This shared inflammatory pathway supports the notion that increased disease activity in AD may predispose to asthma exacerbations due to an overall upregulated Th2 response. Asthma severity was similar in patients with and without concomitant AD, but daily asthma symptoms were associated with not having AD. Asthma severity, according to GINA, reflects treatment intensity rather than symptom control. A higher proportion of patients with concomitant AD reported dupilumab treatment, a therapy targeting shared type-2 inflammatory pathways in both diseases, which may be relevant when interpreting differences in symptom control.

AD involvement of the head-and-neck region and the hands, both considered high-burden skin areas, was common (54.0%, 61.3%) and associated with higher PO-SCORAD in fully adjusted analyses, indicating that some areas, independent from affected BSA, are associated with more severe AD. These estimates are higher than those from our recent AD cohort, where 44.3% and 51.3% had HND and HE, respectively (32). In the cohort, HND and HE were also associated with increasing AD severity when adjusted for BSA (32). This suggests that widespread AD involves specific anatomical sites, defining a distinct endotype characterized by more severe AD. However, as the PO-SCORAD also incorporates sleep and itch and is evaluated by the patients themselves, the association also reflects an increased subjective disease burden. We have shown that HND was associated with asthma, and pediatric and adult studies have shown that filaggrin gene (FLG) mutations are associated with AD in the head-and-neck area as well as respiratory disease, including asthma (41–45). HE has also been associated with FLG mutations in patients with an AD history (46). This may reflect barrier-related effects of FLG mutations across skin and airways, resulting in a subgroup with respiratory comorbidity and anatomically specific disease. Evidence on foot involvement is scare, as most studies have examined it in the context of concomitant HE, although FLG mutation carriers, who have the most severe AD forms, also have foot eczema more frequently (47–49). A US study of 602 AD adults reported that 17.8.1%–28.5% had active AD of the feet (50). In our study, foot involvement was reported by 14.7%, who had high PO-SCORAD scores; however, it was not independently associated with higher AD severity, suggesting that these patients also had AD in high-burden areas or large BSA involvement.

### Strengths and limitations

Patients with asthma and those with concomitant AD were identified in the NPR using validated ICD-10 codes (29, 51), supporting population validity. Despite the low response rate, which may introduce bias and reduce generalizability, and our inability to assess whether non-responders differed from responders in terms of socioeconomic characteristics, the cohort included 4,126 adults with asthma. Asthma and AD severity were evaluated using GINA and PO-SCORAD, both well-established tools. However, PO-SCORAD relies on self-assessment, potentially introducing variability compared with clinician-rated measures (21). The study examined AD in asthma patients; however, asthma with concomitant AD and AD with concomitant asthma represent the same disease overlap from different perspectives. Outcomes were survey-based, potentially introducing reporting bias. Self-reported data captured medications actually taken, enabling assessment of asthma severity based on real-world medication use. Female participation predominated, leading to higher representation of women, which is in line with previous data (52, 53). Therefore, the high proportion of women with both asthma and AD should be interpreted with caution, and this could be influenced by selection bias. Data derived from a predominantly white population limits applicability to other skin types.

## Conclusion

Adults with asthma and concomitant AD shared characteristics of AD cohorts. The AD prevalence of 5.2% was high, and most patients with coexisting disease had childhood onset of both conditions, were women, and exhibited mild-to-moderate AD. Involvement of the head-and-neck region and the hands was frequent and associated with more severe AD, as was the occurrence of AD flares during asthma exacerbation. These findings underscore the need for clinical vigilance in identifying persistent or recurrent disease and highlight the importance of improved recognition of asthma patients with mild-to-moderate AD to ensure adequate referral, as site-specific involvement may indicate more severe AD.

## Data Availability

The datasets presented in this article are not readily available because the data analyzed in this study were obtained from nationwide health registries and contain sensitive personal information. Due to legal and ethical restrictions related to participant confidentiality and data protection regulations, the underlying individual-level data cannot be made publicly available, as this could compromise the anonymization of participants. However, aggregated results and specific outcomes for predefined variables can be made available by the corresponding author upon reasonable request, provided that the request complies with applicable data protection legislation and relevant data access regulations. Requests to access the datasets should be directed to the corresponding author.
