# Supplementary material for: Clinical characteristics and severity of concomitant atopic dermatitis in adults with asthma: a nationwide population-based registry study
Source: Front Allergy. 2026 Jun 11;7:1826356. doi: 10.3389/falgy.2026.1826356 (PMC13294222; doi:10.3389/falgy.2026.1826356)
Supplement: Supplementary file 1 [file Table1.docx]

**SUPPLEMENTARY MATERIAL**

**Supplementary table 1 –** Assessment of signs and symptoms of asthma and atopic dermatitis through the Danish National Patient Register and a nationwide online questionnaire.

|  | **Assessment / Question** |  |
| --- | --- | --- |
| **Asthma**   - ICD-10 code J45.X in the Danish National Patient Register | | |
| **Age at asthma onset** | How old were you when you were diagnosed with asthma? | - 6 years or younger - 7-10 years - 11-15 years - 16-18 years - 19-29 years - 30 years or older - Don’t know / don’t remember |
| **Asthma symptoms the last 12 months** | Have you had asthma symptoms within the past 12 months? | - Yes - No - Don’t know / don’t remember |
| **Asthma severity** | The global Initiative for Asthma (GINA) 2024 guidelines – Step 1-5:  Defined according to current medication use the last four weeks.  Step 1-2: Mild asthma  Step 3: Moderate asthma  Step 4: Moderate-to-severe asthma  Step 5: Severe asthma | |
| **Asthma control**  The Asthma Control Questionnaire-5 (ACQ-5)  Prednisolone use for asthma the last 12 months  Asthma-related night wakings the last 4 weeks | Adequately controlled asthma was defined as an ACQ-5 score below 0.75.  A score between 0.75 and 1.25 indicates borderline adequate control, while a score above 1.25 is said to be not well controlled  Have you been treated with prednisolone tablets (corticosteroids) because of your asthma within the past 12 months?  If you think back over the past 4 weeks, have you woken up at night because of your asthma? | - Yes - No - Don’t know / don’t remember - No, I have not woken up at night because of asthma - Yes, I have woken up at night because of asthma |
| **Asthma-related quality of life** | The Asthma Quality of Life Questionnaire (AQLQ).  A questionnaire with 32 items distributed into four domains. A score can range from 1-7 with higher scores indicating better quality of life. | |
| **Atopic dermatitis** | | |
| Atopic dermatitis onset | How old were you when a doctor first told you that you had atopic eczema? | - 2 years - 2-5 years - 6-10 years - 11-18 years - Over 18 years - Don’t know / can’t remember |
| Atopic dermatitis severity | The Patient-Oriented SCORing Atopic Dermatitis (PO-SCORAD)  The Patient-Oriented Eczema Measure (POEM) | |
| Quality of life | The Dermatology Life Quality Index (DLQI) | |

**Supplementary table 2 –** Age and sex distribution in the invited and participating population of asthma patients identified through the Danish National Patient Register.

|  | **Invited^*^, n** | **Invited, %** | **Participating, n** | **Participating, %** | **Response rate in each age and sex group, %** |
| --- | --- | --- | --- | --- | --- |
| *All asthma patients* | 27,664 | 100.0 | 4,126 | 100.0 | 14.9 |
| *Women, overall* | 17,840 | 64.5 | 2,899/4,126 | 70.3 | 16.3 |
| *Women, 18-30 years* | 2,992 | 10.8 | 481/4,126 | 11.7 | 16.1 |
| *Women, >30-45 years* | 14,846 | 53.7 | 2,417/4,126 | 58.6 | 16.3 |
| *Men, overall* | 9,824 | 35.5 | 1,227/4,126 | 29.7 | 12.5 |
| *Men, 18-30 years* | 1,587 | 5.7 | 145/4,126 | 3.5 | 9.1 |
| *Men, >31-45 years* | 8,237 | 29.8 | 1,081/4,126 | 26.2 | 13.1 |

Abbreviations: n=number.
*2 patients missing data regarding age.

**Supplementary table 3 –** Overview of current asthma treatments in patients with and without concomitant atopic dermatitis.

|  | **Asthma patients with AD (n=214)** | **Asthma patients without AD (n=3,912)** | **All asthma patients (n=4,126)** |
| --- | --- | --- | --- |
| % (n/n total) |  |  |  |
| **SABA** | **40.2** (86/214) | **33.1** (1,293/3,912) | **33.4** (1,379/4,126) |
| **LTRA** | **0.0** (0/214) | **<0.08** (<3/3,912) | **<0.07** (<3/4,126) |
| **ICS** | **21.0** (45/214) | **17.6** (687/3,912) | **17.7** (732/4,126) |
| **ICS/LABA** | **35.5** (76/214) | **37.6** (1,472/3,912) | **37.5** (1,548/4,126) |
| **ICS/LABA/LAMA** | **<1.4** (<3/214) | **<1.9** (<73/3,912) | **2.8** (73/4,126) |
| **Biologics** | **<1.4** (<3/214) | **<0.7** (<29/3,912) | **0.7** (29/4,126) |

Abbreviations: AD, atopic dermatitis; ICD-10, the International Classification of Diseases 10^th^; ICS, inhaled corticosteroids; ICS/LABA, inhaled corticosteroids/long-acting beta_2_-agonist; ICS/LABA/LAMA, inhaled corticosteroids/long-acting beta_2_-agonist/long-acting muscarinic antagonist; LTRA, leukotrine receptor antagonist; n, number; SABA, short-acting beta_2_-agonist.
Biologics: defined as either current use of reslizumab, dupilumab, benralizumab, mepolizumab, or omalizumab.

**Supplementary table 4 -** PO-SCORAD and POEM stratified by different clinical characteristics in patients with a history of hospital-recorded AD.

|  |  | **With hospital-recorded AD (n=214)** | |
| --- | --- | --- | --- |
|  | | **PO-SCORAD, mean (SD)** | **POEM, mean (SD)** |
| **Sex** | **Male** | 27.7 (17.6) | 9.0 (7.1) |
|  | **Female** | 24.6 (15.6) | 8.9 (6.6) |
| **Age** | **18-30 years** | 28.9 (15.3) | 12.2 (7.5) |
|  | **>30 years** | 24.4 (16.2) | 8.5 (6.4) |
| **Onset of AD** | **Childhood** | 25.8 (15.9) | 9.0 (6.6) |
|  | **Adult** | 21.4 (18.4) | 8.4 (7.2) |
| **Flare of AD during asthma exacerbation** | **Yes** | 38.6 (13.5) | 13.6 (5.3) |
|  | **No** | 21.4 (13.9) | 7.3 (5.7) |
| **Head-and-neck dermatitis** | **Yes** | 33.0 (14.8) | 12.3 (5.8) |
|  | **No** | 16.4 (11.8) | 4.8 (5.2) |
| **Hand eczema** | **Yes** | 31.1 (14.4) | 11.7 (5.5) |
|  | **No** | 16.2 (14.4) | 4.5 (5.5) |
| **Genital eczema** | **Yes** | 36.3 (18.3) | 16.4 (5.9) |
|  | **No** | 25.0 (18.3) | 8.7 (6.6) |
| **Foot eczema** | **Yes** | 38.4 (15.0) | 13.9 (5.6) |
|  | **No** | 23.1 (15.2) | 8.1 (6.5) |
| ***Asthma characteristics*** | | | |
| **Onset of asthma** | **Childhood** | 26.0 (16.3) | 9.5 (6.6) |
|  | **Adult** | 22.3 (15.1) | 7.9 (6.6) |
| **Asthma symptoms last 12 months** | **Yes** | 26.3 (15.7) | 11.4 (5.5) |
|  | **No** | 23.9 (15.7) | 9.8 (6.4) |
| **GINIA** | **Step 1-2 (Mild)** | 25.3 (17.6) | 8.8 (6.9) |
|  | **Step 3 (Moderate)** | 24.0 (13.9) | 8.4 (5.9) |
|  | **Step 4 (Moderate-to-Severe)** | 28.3 (17.0) | 9.8 (6.7) |
|  | **Step 5 (Severe)** | 25.5 (17.8) | 10.8 (8.3) |
| **ACQ-5** | **<= 0.75 (Well controlled)** | 24.5 (16.2) | 8.8 (6.7) |
|  | **>0-75 (Partially to uncontrolled)** | 27.3 (15.9) | 9.3 (6.6) |
| **Daily asthma symptoms >2 days per week** | **Yes** | 28.3 (14.7) | 9.9 (6.8) |
|  | **No** | 24.7 (16.4) | 8.7 (6.6) |
| **Asthma-related night awakenings the last 4 weeks** | **Yes** | 27.7 (18.2) | 9.7 (8.4) |
|  | **No** | 25.1 (15.9) | 8.9 (6.5) |
| **Systemic prednisolone for asthma the last 12 months** | **Yes** | 31.6 (16.9) | 11.3 (6.7) |
|  | **No** | 25.7 (15.6) | 9.4 (6.6) |
| **Emergency room or hospitalisation the last 12 months due to asthma** | **Yes** | 24.7 (16.6) | 12.2 (8.0) |
|  | **No** | 26.3 (15.7) | 9.4 (8.0) |
| **AQLQ score** | **1-5 (Worse quality of life)** | 29.7 (16.7) | 9.6 (7.4) |
|  | **6-7 (Better quality of life)** | 24.0 (15.9) | 8.8 (6.6) |

Abbreviations: ACQ-5, the Asthma Control Questionnaire-5; AD, atopic dermatitis; AQLQ, the Asthma quality of life questionnaire; ICD-10, the International Classification of Diseases 10^th^; GINA, the Global Initiative for Asthma; PO-SCORAD, the Patient-Oriented SCORing Atopic dermatitis, POEM, the Patient-Oriented Eczema Measure; SD, standard deviation.
Overview of mean (SD) PO-SCORAD, and mean (SD) POEM score in asthma patients with self-reported physician-diagnosed AD and hospital-recorded AD (ICD-10, L20.X).

**Supplementary table 5 –** Atopic dermatitis-related characteristics associated with increasing PO-SCORAD.

|  |  | **PO-SCORAD, adjusted β [95% CI]** |
| --- | --- | --- |
|  |  | **Model 1** |
| **Age** | | -0.17 [-0.54-0.20] |
| **Sex** | **Man** | Ref |
|  | **Women** | -2.11 [-6.7-2.43] |
| **Onset of AD** | **Childhood** | 2.40 [-4.19-9.00] |
|  | **Adult** | Ref |
| **High-burden skin areas of AD** | **Head-and-neck** | 5.94 [0.34-11.60]* |
|  | **Hands** | 8.37 [4.07-12.70]*** |
|  | **Genitals** | -2.25 [-13.40-8.88] |
| **Foot involvement of AD** | | 0.48 [-6.18-7.14] |
| **AD affected BSA** | **<10** | Ref |
|  | **10-40** | 8.06 [2.12-14.0]** |
|  | **>40** | 23.7 [13.9-33.5]*** |

Abbreviations: AD, atopic dermatitis; BSA, body surface area; PO-SCORAD, the Patient-Oriented SCORing Atopic Dermatitis; 95% CI, 95% confidence interval.

Multiple linear regression model showing the association between different AD-related characteristics and AD severity according to PO-SCORAD.

*p<0.05, **p<0.01, ***p<0.001

**Supplementary table 6 –** Characteristics associated with AD according to a TCS/TCI prescriptions the previous 12 months, adjusted odds ratio and 95% confidence interval.

|  |  | **A prescription for TCS and or TCI the previous 12 months, aOR [95% CI]** | | |
| --- | --- | --- | --- | --- |
|  |  | **Model 1^a^** | **Model 2^b^** | **Model 3^c^** |
| **Age** | | 0.99 [0.97-1.00] | 0.99 [0.97-1.00] | 0.99 [0.97-1.00] |
| **Sex** | **Men** | Ref | Ref | Ref |
|  | **Women** | 0.91 [0.75-1.13] | 1.92 [0.75-1.13] | 0.90 [0.73-1.11] |
| **Educational level** | **Low** | Ref | Ref | Ref |
|  | **Middle** | 1.15 [0.90-1.47] | 1.15 [0.90-1.47] | 1.16 [0.91-1.49] |
|  | **High** | 1.09 [0.83-1.42] | 1.08 [0.83-1.42] | 1.09 [0.83-1.44] |
| **Asthma severity, GINA** | **Mild** | Ref | Ref | Ref |
|  | **Moderate** | 1.17 [0.95-1.66] | 1.17 [0.95-1.44] | 1.16 [0.93-1.44] |
|  | **Moderate-to-severe** | 1.26 [0.95-1.66] | 1.26 [0.95-1.66] | 1.26 [0.94-1.68] |
|  | **Severe** | 1.11 [0.78-1.55] | 1.11 [0.78-1.56] | 1.11 [0.77-1.58] |
| **Onset of asthma** | **Childhood** |  | 1.05 [0.86-1.28] | 1.04 [0.85-1.26] |
|  | **Adult** |  | Ref | Ref |
| **ACQ-5 score** | **Uncontrolled** |  |  | 1.04 [0.84-1.27] |
|  | **Well controlled** |  |  | Ref |

Abbreviations: aOR, adjusted odds ratio; ACQ-5, the Asthma Control Questionnaire-5; AD, atopic dermatitis; GINA, the Global Initiative for Asthma; 95% CI, 95% confidence interval.

Logistics regression models showing clinical characteristics associated with a prescription of TCS and or TCI the previous 12 months in asthma patients. ^a^=In model 1 age (continuous), sex (men/women), educational level (low/middle/high), and asthma severity according to GINA (mild/moderate/moderate-to-severe/severe) are included as explanatory variables. ^b^=In model 2 age (continuous), sex (men/women), educational level (low/middle/high), asthma severity according to GINA (mild/moderate/moderate-to-severe/severe), and age of asthma onset (childhood/adulthood) are included as explanatory variables. ^c^=In model 3 age (continuous), sex (men/women), educational level (low/middle/high), asthma severity according to GINA (mild/moderate/moderate-to-severe/severe), age of asthma onset (childhood/adulthood), and asthma control according to the ACQ-5 score (not weel controlled/well controlled) are included as explanatory variables.

***: p<0.001

**Figure Legend – Supplementary Figure 1:**

**Supplementary figure 1** – Prevalence of asthma patients who had a history of atopic dermatitis stratified by A) asthma severity, and B) age.

Abbreviations: ICD-10, the International Classification of Diseases 10^th^; GINA, the Global Initiative for Asthma.
Bar plot showing the prevalence of asthma patients who had a history of hospital-recorded (ICD-10 L20.X) atopic dermatitis stratified by asthma severity according to the GINA guidelines and age.
