# Supplementary figures and images for: Clinical characteristics and severity of concomitant atopic dermatitis in adults with asthma: a nationwide population-based registry study
Source: Front Allergy. 2026 Jun 11;7:1826356. doi: 10.3389/falgy.2026.1826356 (PMC13294222; doi:10.3389/falgy.2026.1826356)

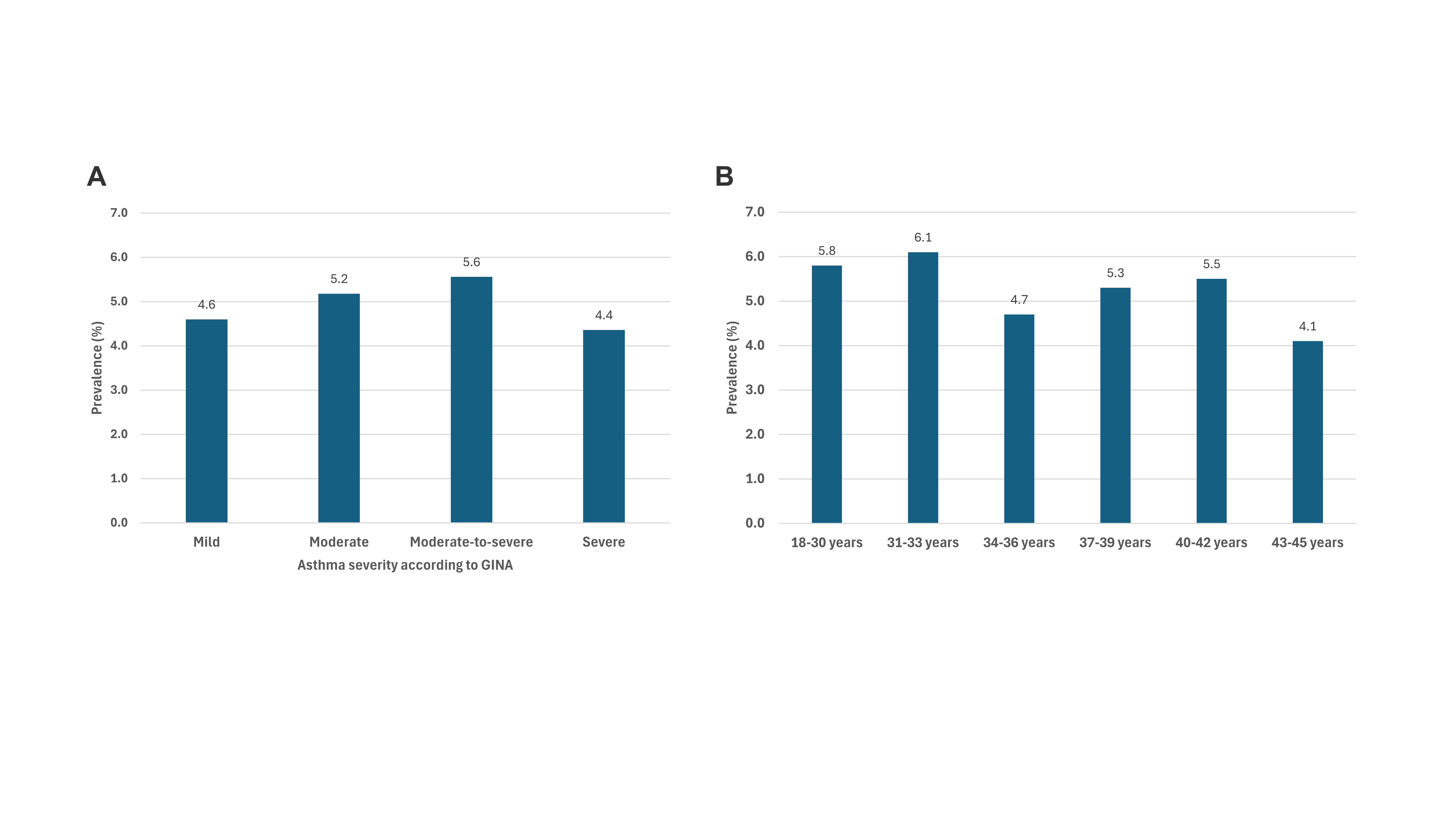

Supplement: Supplementary file 2 [file image1.tif]
